# The treatment of patellar tendinopathy

**DOI:** 10.1007/s10195-012-0220-0

**Published:** 2012-12-28

**Authors:** E. C. Rodriguez-Merchan

**Affiliations:** 1 Department of Orthopaedic Surgery, La Paz University Hospital, Madrid, Spain; 2 School of Medicine, Autonomous University, Madrid, Spain

**Keywords:** Patellar tendinopathy, Treatment, Conservative, Surgical

## Abstract

**Background:**

Patellar tendinopathy (PT) presents a challenge to orthopaedic surgeons. The purpose of this review is to revise strategies for treatment of PT

**Materials and methods:**

A PubMed (MEDLINE) search of the years 2002–2012 was performed using "patellar tendinopathy" and "treatment" as keywords. The twenty-two articles addressing the treatment of PT with a higher level of evidence were selected.

**Results:**

Conservative treatment includes therapeutic exercises (eccentric training), extracorporeal shock wave therapy (ESWT), and different injection treatments (platelet-rich plasma, sclerosing polidocanol, steroids, aprotinin, autologous skin-derived tendon-like cells, and bone marrow mononuclear cells). Surgical treatment may be indicated in motivated patients if carefully followed conservative treatment is unsuccessful after more than 3–6 months. Open surgical treatment includes longitudinal splitting of the tendon, excision of abnormal tissue (tendonectomy), resection and drilling of the inferior pole of the patella, closure of the paratenon. Postoperative inmobilisation and aggressive postoperative rehabilitation are also paramount. Arthroscopic techniques include shaving of the dorsal side of the proximal tendon, removal of the hypertrophic synovitis around the inferior patellar pole with a bipolar cautery system, and arthroscopic tendon debridement with excision of the distal pole of the patella.

**Conclusion:**

Physical training, and particularly eccentric training, appears to be the treatment of choice. The literature does not clarify which surgical technique is more effective in recalcitrant cases. Therefore, both open surgical techniques and arthroscopic techniques can be used.

## Introduction

There is agreement within the literature that the patellar tendon is particularly vulnerable to injury and often difficult to manage successfully [1–5]. The pathological process of patellar tendinopathy (PT) includes various aspects. Inflammation was believed central to the pathologic process, but histopathologic evidence has confirmed the failed healing response nature of these conditions [2]. Excessive or inappropriate loading of the musculotendinous unit is believed to be central to the disease process, although the exact mechanism by which this occurs remains uncertain. Additionally, the location of the lesion (for example, the midtendon or osteotendinous junction) has become increasingly recognized as influencing both the pathologic process and subsequent management.

Danielson et al. [6] found a marked sympathetic component in the perivascular innervation of the dorsal paratendinous tissue of the patellar tendon in arthroscopically treated patients. The study demonstrated, for the first time, the innervation patterns of the area dorsal to the patellar tendon in man. It showed that the area investigated is under marked influence by the sympathetic nervous system. Thus, sympathetic effects are likely to occur for blood vessels of the area, which is interesting since color Doppler has revealed that vessels of this area (“neovessels”) display a pathologically high blood flow in tendinosis.

The purpose of this review is to revise strategies for the treatment of PT.

## Materials and Methods

PubMed articles (MEDLINE) in English related to the treatment of PT were searched, using “patellar tendinopathy” and “treatment” as key words. Between 2002 and 2012, we found 186 references. We chose the 22 references that had the higher level of evidence and that were closely related to the treatment of PT.

## Results

There are several strategies for the management of PT: therapeutic exercises, extracorporeal shock wave therapy (ESWT), injections, open surgical procedures and arthroscopic techniques. It is commonly accepted that surgical treatment must be indicated in motivated patients if carefully followed conservative treatment (physical training, injections, ESWT) is unsuccessful after 3–6 months [12–22].

### Therapeutic exercises

Hyman studied PT in volleyball athletes [3]. He found that PT affected nearly one half of elite volleyball athletes and caused significant morbidity. His study showed that conservative treatment was very effective using an eccentric exercise regimen and decline squats [2, 3]. Physical training, and particularly eccentric training, has been reported to be the treatment of choice for patients suffering from PT [7].

### Extracorporeal shockwave therapy

ESWT appears to be a promising treatment in patients with chronic PT. ESWT is most often applied after the eccentric training has failed. Zwerver et al. [8] studied the effectiveness of ESWT in athletes with PT who were still in training and competition. They performed a randomized study, and compared ESWT versus placebo in patients with symptoms of PT of 3–12 months duration. The only benefit found was a subjective improvement. Other objective parameters did not improve.

### Injection treatments

Injection treatments are increasingly used as treatment for PT. Van Ark et al. [9] described different injection treatments: platelet-rich plasma (PRP), sclerosing polidocanol, steroids and aprotinin. All the different injection treatments seem promising for treating PT. Unlike the other injection treatments, steroid treatment often showed a relapse of symptoms in the long term. These results, however, should be interpreted with caution, as the number of studies is low, few high-quality studies have been conducted and the studies are hard to compare due to different methodology.

Ultrasound-guided injection of autologous skin-derived tendon-like cells has been show to be more effective than plasma alone for the treatment of refractory PT [10].

Pascual-Garrido et al. [11] tried to determine if patients with chronic PT will improve clinically after the inoculation of bone marrow mononuclear cells (BM-MNCs). Eight patients with chronic PT were included. Patients averaged 24 years old (range 14–35 years). All patients were refractory to conservative treatment for at least 6 months before the procedure. BM-MNCs were harvested from the iliac bone crest and inoculated under ultrasound guide in the patellar tendon lesion. Improvement was assessed through established clinical scores and ultrasound. At 5-year follow-up, seven of eight patients said they would have the procedure again if they had the same problem in the opposite knee and were completely satisfied with the procedure. Seven of eight patients thought that the results of the procedure were excellent. According to these results, inoculation of BM-MNCs could be considered as a potential therapy for those patients with chronic PT refractory to nonoperative treatments.

### Open surgical treatment

Ferreti et al. [12] analyzed the results at a minimum of 5 years after the performance of a surgical technique in competitive athletes. Thirty-two patients (thirty-eight knees) affected by PT were treated surgically after failure of nonoperative treatment. All knees were operated on by the same surgeon using the same surgical technique: longitudinal splitting of the tendon, excision of any abnormal tissue that was identified, and resection and drilling of the inferior pole of the patella. The result was excellent in twenty-three knees (70 %), good in five, fair in one, and poor in four at the time of the long-term follow-up. Eighty-two percent of the patients who tried to pursue sports at their preinjury level were able to do so, and 63 % of those knees were totally symptom-free. The outcome of the reported surgical treatment appeared to be satisfactory; however, the results were less predictable in volleyball players.

Kaeding et al. [4] found a 71 % success rate when open surgical treatment of the inferior pole of the patella was performed, compared to 92 % when no patella bony work was carried out. Closure of the paratenon showed a 85 % success rate compared to 91.5 % when no paratenon closure was performed. Immobilization showed a 82.5 % success rate compared to 95 % success when no postoperative immobilization was used.

Shelbourne et al. [13] reported that surgical removal of necrotic tissue, surgical stimulation of remaining tendon, and aggressive rehabilitation after patellar tendonectomy could allow athletes to return to sports. Overall, tendonectomy, surgical tendon stimulation, and aggressive postoperative rehabilitation were found to be a safe, effective way to return high-level athletes to their sports.

### Arthroscopic treatment

Fifteen patients with PT were treated by Wilberg et al. [14]. All patients were treated with arthroscopic shaving of the dorsal side of the proximal tendon. The short-term results of this study indicated that arthroscopic shaving targeting the area with neovessels and nerves on the dorsal side of the patellar tendon has a potential to reduce the tendon pain and allow for the majority of patients to go back to full tendon loading activity within 2 months after surgery.

Ogon et al. [15] described an arthroscopic technique for the treatment of chronic PT. Diagnostic arthroscopy was performed and hypertrophic synovitis around the inferior patellar pole was removed with a bipolar cautery system. Two outside-in cannulas marked the clinically symptomatic region, mainly found between the tendon insertion site and the lateral aspects of the patellar tendon. The bipolar cautery was used for a release of the paratenon and a bone denervation at the inferior patellar pole including the tendon insertion site within the marked area. No tendon or bone material was removed or excised throughout the procedure. The minimal surgical impact to the tendon allowed early and functional rehabilitation. The technique was effective, easy to perform, and safe to apply.

Kelly examined the results of arthroscopic tendon debridement with excision of the distal pole of the patella for refractory PT [16]. He concluded that arthroscopic excision of the distal patellar pole with tendon debridement holds promise for the treatment of refractory PT.

Lorbach et al. [17] performed a prospective study to evaluate the clinical results of arthroscopic resection of the lower patellar pole in patients with PT. The main conclusion was that arthroscopic resection of the lower patellar pole as a minimal invasive method to treat PT provides satisfactory clinical results in knee function and pain reduction with fast recovery and return to sport activities.

Pascarella et al. [18] analyzed medium-term and long-term outcome of patients undergoing arthroscopic surgery for the management of PT after failing nonoperative treatment. All patients were able to return to sports by 3 months. Arthroscopic surgery for patients with PT, refractory to nonoperative management, appears to provide significant improvements in symptoms and function, with improvements maintained for at least 3 years. These results suggested that some patients may not be able to achieve their presymptom sporting level; or if they do, they may participate in sports with some degree of residual symptoms. Limited data show that these improvements are maintained for up to 10 years. Early return to sports may also be achieved.

Bayar et al. [19] reported on the fate of patellar tendon and infrapatellar fat pad after arthroscopy via central portal. Central patellar portal is an accessory portal in arthroscopic knee surgery, which generally is considered to be safe. The study showed that although central patellar portal did not cause any clinical problems in a low demand group of patients, it leads to a significant radiological sequela in the tendon, the biomechanical significance of which needs to be clarified.

### Comparative studies

#### Surgery versus eccentric training

Surgical treatment (patellar tenotomy) was compared with eccentric training by Bahr et al. [20]. No advantage was demonstrated for surgical treatment compared with eccentric strength training. They stated that eccentric training should be tried for 12 weeks before open tenotomy is considered for the treatment of PT.

#### Sclerosing polidocanol injections versus arthroscopy

Willberg et al. [21] compared the clinical effects after treatment with sclerosing polidocanol injections and arthroscopic shaving. Fifty-two patellar tendons (43 men and two women) with ultrasound and colour Doppler-verified diagnosis of PT/JK were randomly assigned to treatment with ultrasound and colour Doppler-guided sclerosing polidocanol injections (group A) or ultrasound and colour Doppler-guided arthroscopic shaving (group B). After treatment, the patients treated with arthroscopic shaving had a significantly lower visual analogue score (VAS) score at rest and during activity, and were significantly more satisfied compared with the patients in the sclerosing injection group.

#### Open surgery versus arthroscopic surgery

Cucurulo et al. [22] evaluated the results of arthroscopic procedures in the treatment of PT. A retrospective multicenter study was performed in four centers. Patients were athletes who did not respond to carefully followed conservative treatment and who underwent surgery. Sixty-four patients were included, ten who underwent arthroscopy. The main conclusion was that surgical treatment is indicated in motivated athletes if carefully followed conservative treatment is unsuccessful after more than 6 months, making it impossible to practice a sport. Arthroscopic techniques seemed to be as effective as open surgery, with an equivalent delay for beginning sports activities.

## Discussion

PT is a common, painful, overuse disorder. Although many different treatment methods have been described, there is no consensus regarding the optimal treatment for this condition [1–22].

In this review, no advantage has been demonstrated between surgical treatment and eccentric strength training [21]. Therefore, eccentric training should be tried for 12 weeks before open tenotomy is considered for the treatment of PT. Both sclerosing polidocanol injections and arthroscopic shaving have shown good clinical results, but patients treated with arthroscopic shaving had less pain and were more satisfied with the treatment result [19]. Arthroscopic techniques seemed to be as effective as open surgery [20].

Another review has shown strong evidence for the use of eccentric training to treat PT [7]. There was, however, limited evidence for surgery, sclerosing injections, and ESWT therapy. Physical training, and particularly eccentric training, was reported to be the treatment of choice for patients suffering from PT. However, type of exercise, frequency, load, and dosage require further investigation.

In conclusion, it is commonly accepted that surgical treatment must be indicated in motivated patients if carefully followed conservative treatment (physical training, injections, ESWT) is unsuccessful after 3–6 months [12–22]. The literature, however, does not clarify which surgical technique is more effective. Therefore, both open surgical techniques and arthroscopic techniques can be used. Physical training, and particularly eccentric training, appears to be the treatment of choice for patients suffering from PT.
